# Functional Gait Assessment Using Manual, Semi-Automated and Deep Learning Approaches Following Standardized Models of Peripheral Nerve Injury in Mice

**DOI:** 10.3390/biom12101355

**Published:** 2022-09-23

**Authors:** Daniel Umansky, Kathleen M. Hagen, Tak Ho Chu, Rajesh K. Pathiyil, Saud Alzahrani, Shalina S. Ousman, Rajiv Midha

**Affiliations:** 1 Department of Clinical Neurosciences, Cumming School of Medicine, Hotchkiss Brain Institute, University of Calgary, Calgary, AB T2N 4N1, Canada; 2 Department of Neuroscience, Hotchkiss Brain Institute, University of Calgary, Calgary, AB T2N 4N1, Canada; 3 Department of Clinical Neurosciences and Cell Biology and Anatomy, Hotchkiss Brain Institute, University of Calgary, Calgary, AB T2N 4N1, Canada

**Keywords:** CNN, convolutional neural network, crush injury, deep learning, DNNs, deep neural networks, gait analysis, NIC, neuroma in continuity, NCV, nerve conduction velocity, PNI, peripheral nerve injury, SFI, sciatic functional index, sciatic nerve, semi-automated, stretch–crush injury, VGL, Visual Gait Lab, von Frey monofilament test

## Abstract

Objective: To develop a standardized model of stretch–crush sciatic nerve injury in mice, and to compare outcomes of crush and novel stretch–crush injuries using standard manual gait and sensory assays, and compare them to both semi-automated as well as deep-learning gait analysis methods. Methods: Initial studies in C57/Bl6 mice were used to develop crush and stretch–crush injury models followed by histologic analysis. In total, 12 eight-week-old 129S6/SvEvTac mice were used in a six-week behavioural study. Behavioral assessments using the von Frey monofilament test and gait analysis recorded on a DigiGait platform and analyzed through both Visual Gait Lab (VGL) deep learning and standardized sciatic functional index (SFI) measurements were evaluated weekly. At the termination of the study, neurophysiological nerve conduction velocities were recorded, calf muscle weight ratios measured and histological analyses performed. Results: Histological evidence confirmed more severe histomorphological injury in the stretch–crush injured group compared to the crush-only injured group at one week post-injury. Von Frey monofilament paw withdrawal was significant for both groups at week one compared to baseline (*p* < 0.05), but not between groups with return to baseline at week five. SFI showed hindered gait at week one and two for both groups, compared to baseline (*p* < 0.0001), with return to baseline at week five. Hind stance width (HSW) showed similar trends as von Frey monofilament test as well as SFI measurements, yet hind paw angle (HPA) peaked at week two. Nerve conduction velocity (NCV), measured six weeks post-injury, at the termination of the study, did not show any significant difference between the two groups; yet, calf muscle weight measurements were significantly different between the two, with the stretch–crush group demonstrating a lower (poorer) weight ratio relative to uninjured contralateral legs (*p* < 0.05). Conclusion: Stretch–crush injury achieved a more reproducible and constant injury compared to crush-only injuries, with at least a Sunderland grade 3 injury (perineurial interruption) in histological samples one week post-injury in the former. However, serial behavioral outcomes were comparable between the two crush groups, with similar kinetics of recovery by von Frey testing, SFI and certain VGL parameters, the latter reported for the first time in rodent peripheral nerve injury. Semi-automated and deep learning-based approaches for gait analysis are promising, but require further validation for evaluation in murine hind-limb nerve injuries.

## 1. Introduction

Peripheral nerve injury (PNI) following trauma or neuropathies entails severe long-term disability, rehabilitation and costs. Over 200,000 trauma-related nerve injuries per year occur in the United States alone [1], and over 20 million people are estimated to be affected by peripheral neuropathies [2]; the annual incidence approximates 45 cases per 100,000, similar to the incidence of epilepsy [3,4]. Surgical nerve repair, while feasible, leads to an often-unpredictable process in which recovery and outcome depend on the injury mechanism itself, nerve microarchitecture involvement and the technical aspects of the repair used. Regenerating axons need to overcome injury gaps and the distal denervated nerve environment, in which the chronically denervated Schwann cells cannot support axon degeneration optimally [5,6,7]. Reproducible, unbiased analysis of PNI models in rodents remain a significant obstacle to overcome [8]. PNI models have been previously reported and validated by our group [9,10] in a rat model. Yet, creating a more realistic model which will resemble clinical conditions in mice is still cumbersome [8]. A stretch–crush injury, one of the most commonly clinically encountered PNI [11,12,13], which can better mimic and resemble such an injury and further allow development of such investigations in transgenic mice, is therefore warranted.

Behavioral testing using gait analysis can offer objective result interpretation [14] for experimental rodent PNI models, but has also garnered significant debate among investigators in recent years [7,8,10,15,16]. Specifically, nerve regeneration quantification by means of histologic, morphologic and electrophysiological data can have controversial interpretations, as it may not correlate with behavioral function [17,18]. Moreover, works studying locomotor interlimb coordination rating were originally intended to follow spinal cord injury using a point scale formula by incorporating tail and trunk observations along with paw and toe position [19]. Later studies investigated ‘pure’ limb function using formulated multiple linear regression analysis of sciatic functional index (SFI) along with tibial (TFI) and peroneal (PFI) functional indexes in rodent models [12,17]. Given current limitations and time consumption in gait analyses for PNI research models, especially for paw detection and tracking [20], we, herein, present our utilization of Visual Gait Lab (VGL) software for post-injury behavioral analysis. Preliminary work using this technique based on the DeepLabCut (DLC) technological platform [20,21,22] has shown a feasible, user-friendly interface allowing human gait kinematics and reliable neurobehavioral testing in smaller animals [23]. Moreover, multiple analytic variables, previously overlooked, can now provide additional investigative insight to dynamic changes in animal recovery following PNI. We find that the possibility to use the software’s detector architecture with the utilization of a relatively small number of training images [22] allows a desirable learning transfer capability. These variables include hindlimb internal comparison, hind-to-front limb adjustment and front limb model investigation for a combined animal stance and gait tracking over time.

The purpose of the current work is to present a reliable and reproducible model of stretch–crush injury in mice that emulates clinical conditions of a neuroma in continuity (NIC) more closely [9]. Moreover, by evaluating this injury model using a platform of semiautomated methods, we aim to provide additional tools for the research community. We identify the utility of our current model in providing a clear and distinct sciatic nerve injury and demonstrate that nerve regeneration and functional recovery can be characterized using VGL semi-automated approaches.

## 2. Materials and Methods

### 2.1. Animals

Preliminary work was performed on several different groups of mice to determine the optimal configuration for both nerve injury methods (Table 1). In addition to the exploratory phase (development of the model), an acute phase was also assessed in which 4 C57/Bl6 mice were used; one mouse received a crush injury on one leg and a stretch–crush injury on its contralateral leg, while three additional mice received bilateral stretch–crush injuries (detailed below) and were allowed to recover for one week before histological analyses were performed. Following the exploratory and acute stages, 12 (6 per injury condition) female 8-week-old 129S6/SvEvTac mice were used in a behavioural study. All mice were bred and maintained in the University of Calgary, and all protocols carried out were in accordance with the guidelines of the Canadian Council of Animal Care and received approval by the University of Calgary Animal Resources and Ethics Committee (Project AC18-0125). Mice were kept on a 12 h light/dark cycle and had access to food and water ad libitum. Surgeries and electrophysiological readings were carried out under 2–2.5% isoflurane inhalation anesthetic.

### 2.2. Nerve Injury Model

Surgical technique and crush injury methods have been validated in previous work by our group [9] and are presented briefly in the following section. All procedures were performed on hindlimbs, following shaving, along with 70% ethanol aseptic preparation. Animals were kept on top of heating pads to maintain their body temperature until the end of procedures. Surgical procedures were performed under a Leica M651 operating microscope and intraoperative images captured with a microscope camera (Infinity1, Lumenera, Ottawa, ON, Canada). Exposure of the left sciatic nerve (in the case of the behavioural stage; bilaterally for the exploratory and acute stages) was performed via a longitudinal lateral thigh incision, and the sciatic nerve was exposed from its emergence from the sciatic notch to the trifurcation at the popliteal fossa. Retractors were used to keep the area exposed. Injuries were performed 10 mm proximal to the sciatic nerve trifurcation. Mice were randomly assigned to receive either a crush injury (N = 6; achieving a Sunderland grade 2 injury) [9], or a stretch–crush injury (N = 6; attempting to achieve a Sunderland grade 3–4 injury). The specific protocol for each of the injury conditions is detailed below.

#### 2.2.1. Crush Injury

**Figure 1:**
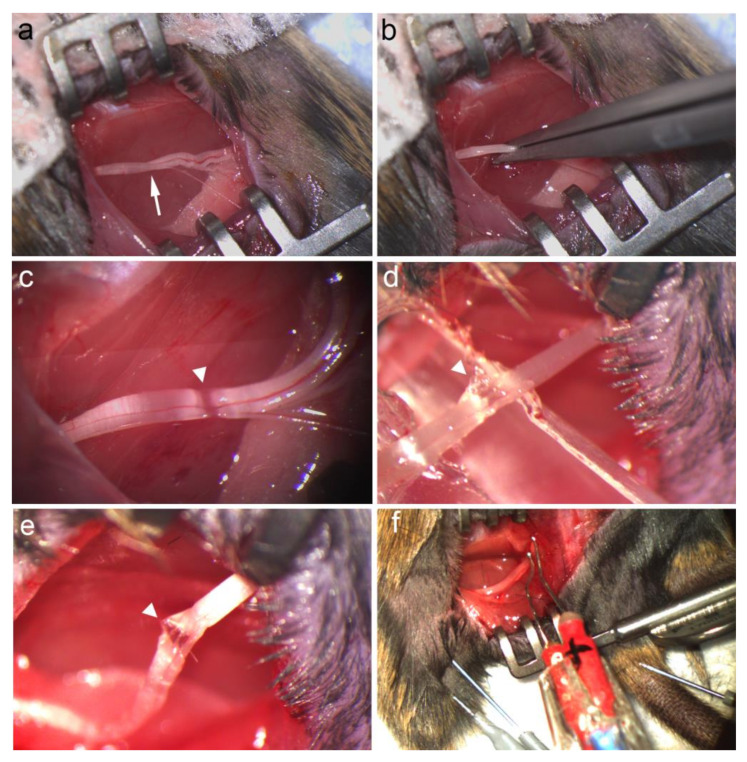
Representative surgical exposure for crush injury method and CMAP recording. (**a**) Exposure of the sciatic nerve (white arrow shows measured crush site 10 mm proximal to the sciatic nerve trifurcation point). (**b**) Crushing the sciatic nerve using #5 jewelers’ forceps applying constant force in a vertical direction for 30 s, followed by horizontal compression for an additional 30 s. (**c**) Verification of crush injury site with preserved nerve continuity (white arrowhead). (**d**) Custom spacer designed to implement 30 g of stretch to the nerve at the predetermined notch while crush is simultaneously performed (white arrowhead stretch–crush site). (**e**) Stretch–crush injury site (white arrowhead) after custom spacer removal; note that nerve continuity is preserved. (**f**) Bipolar direct stimulation and recording electrodes in gastrocnemius muscle setup for NCV and CMAP recording. CMAP, compound muscle action potential; NCV, nerve conduction velocity.

Using fine flat #5 jewelers’ forceps applying a continuous force, the sciatic nerve was crushed for 30 s, then the forceps were removed from the nerve, rotated 90° and then a second crush was performed at the same location to ensure maximal axonal damage, a paradigm which reliably reproduces at least a Sunderland grade 2 injury [9]. The opposite leg served as an injured positive control in the behavioural experiment. The crush site was verified visually (Figure 1) followed by irrigation with normal saline, the removal of the tissue retractors, and the approximation of the biceps femoris muscle. The skin incision was then closed with single horizontal 6–0 Prolene sutures.

#### 2.2.2. Stretch–Crush Injury

Preliminary experiments were performed in the exploratory stage to determine the optimal amount of stretch applied to the sciatic nerve to prevent rupture. Using a pen-style 100 × 1 g traction spring scale applying 30 g of force, the authors measured the distance from the surface of the skin to the point at which the nerve was hooked onto the spring scale (i.e., 3.27 mm; data not shown). In order to accommodate a crush injury, the authors used the degree of elevation of the nerve to develop a custom-made spacer to place beneath the nerve in order to achieve a consistent 30 g stretch (as determined by distance, the nerve was lifted above the skin (i.e., 3.27 mm)) with a notch for a crush to be performed (Figure 1).

For the stretch–crush injury, the spacer was placed under the nerve to elevate it above the skin and was located such that the notch of the spacer lined up with the same site as in the crush injury condition (i.e., 10 mm proximal to the sciatic nerve trifurcation point). Using fine flat #5 jewelers’ forceps applying a continuous force, the sciatic nerve was crushed at the site of the spacer notch for 30 s, and then the forceps were removed from the nerve, rotated 90°, and a second crush was performed at the same location, followed by removal of the spacer. Similar to the crush injury, the stretch–crush site was verified visually, followed by irrigation with normal saline, the removal of the tissue retractors and the approximation of the biceps femoris muscle. Skin incision was then closed with single horizontal 6–0 Prolene sutures. Buprenorphine was provided at a dose of 0.05 mg/kg just prior to, and then the day following surgery, for perioperative analgesia to all mice.

### 2.3. Behavioral Assessments: Von Frey Monofilament Test, Gait Analysis, and SFI

Behavioral assessments were performed on 12 female mice on 2 occasions prior to surgery (i.e., baseline) and weekly for 6 weeks following injury.

#### 2.3.1. Von Frey Monofilament Testing

Quantitative allodynia assessment was performed using a previously reported and validated technique for larger rodents [24,25], later adapted and standardized to estimate paw withdrawal threshold using a simplified up-and-down method [11,26] and its incorporation into mice models utilized [8]. Similar testing has been used for hypesthesia in prior reports [27] and its utilization in mice is still, therefore, limited as described in the discussion of the present paper. Prior to surgery, mice were habituated to the von Frey monofilament test. They were placed into a 4-walled chamber with a grated floor and allowed to acclimatize for several minutes. The experimenter took a number 5 von Frey Filament (EXACTA Precision and Performance monofilaments, Stoelting, Wood Dale, IL, USA) [27] applying a ~9.06 g force, and applied the filament to the plantar surface of the hind paw until the filament began to bend, and marked whether the mouse reacted to the filament (e.g., lifted, licked or shook their paw) over 5 repetitions. If the mouse responded to the filament 3 out of 5 times, then the experimenter switched to the next thinnest filament (i.e., the number 4 filament, applying a ~7.19 g force) and repeated the 5-repetition trial. If the mouse did not respond to the filament 3 out of 5 times, then the experimenter switched to the next thickest filament (i.e., from filament 5, the experimenter would change to filament 6, applying a ~11.40 g force). For each hind paw there were 5 trials (i.e., 5 pokes for each of 5 filaments), unless there was no reaction to any of the filaments, which did occur one week after injuries were performed and sensitivity was low; in this case the filaments were increased until a reaction was found.

#### 2.3.2. Gait Analysis

Following von Frey monofilament testing, mice were transferred to a soundproof room where a DigiGait (Mouse Specifics, Inc., Framingham, MA, USA) treadmill was placed. Similar to the von Frey testing, mice were first habituated to the chamber prior to data collection. Mice were placed individually into the clear-walled chamber set on top of the stationary treadmill belt, allowing the mouse to move around and explore the compartment for approximately 2 min. Then, the treadmill was turned on and the speed was incrementally increased until the animal was walking in the appropriate direction.

For data collection, these steps (strides) were repeated, and the speed was increased to 9 cm/s (this speed was chosen in order to accommodate an injured mouse with reduced ambulatory ability), as reported, to significantly limit observations [28,29]. Mice were recorded ventrally from a camera located below the treadmill belt. Once a recording of the animal walking for at least 4 paces was collected, the treadmill was turned off and the mouse was removed from the chamber and placed back into home cage. The treadmill belt was then wiped with 70% ethanol before the next mouse was put into the chamber and a trial repeated.

**Figure 2:**
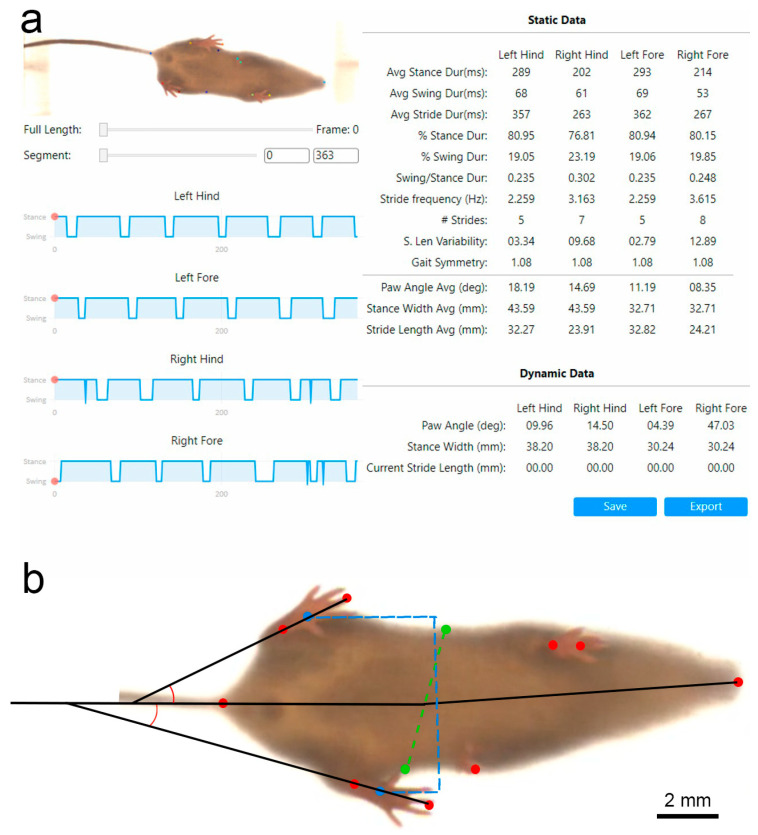
VGL user interface. (**a**) VGL software screenshot. Static and dynamic gait data output window for a representative animal. Software output allows the user to select both static as well as dynamic data on each labelled limb, separately or in comparison to contralateral limb and body position. (**b**) Schematic of markers and measurements of hind stance width (blue dotted line) and paw angles measured in comparison to central body axis used in software. The green dotted line represents body midpoints at abdominopelvic border. Red dots, defined by the user, correspond to body markers of nose, front and hind legs, and rear. See body text for detail. VGL, Visual Gait Lab.

Gait was analyzed using (VGL) [21] for recorded videos of the DigiGait system. Briefly, VGL, based on DLC technological platform [20,21,22], uses a simple and user-friendly graphical interface coupled with the gait analysis system. Following installation of the software, users define markers of interest along the subjected animal image and allow training of the system through a convolutional neural network (CNN). Once training of the system is complete and a desirable 3–5 pixel accuracy is reached—considered the size of a mouse nose [22]—users can evaluate and decide if additional training is required, given by a mean average Euclidean error (MAE), which will ideally be less than the human error variability error [21,22]. Following training, the CNN analysis of videos of interest are performed and gait analysis carried out. Final analysis output is given through static and dynamic data parameters representing gait data points based on averages across a complete video length or a specific video frame viewed by the user, respectively (Figure 2).

#### 2.3.3. Sciatic Functional Index (SFI)

In addition to the VGL analysis of the videos, a blinded manual analysis of 96 gait videos was performed by a non-biased observer. SFI of mice was calculated using the formula described and highly cited previously by Inserra et al. [30]. To obtain the necessary measurements for SFI calculation, 6 screenshots were taken from each video. These specific 6 frames were chosen, where 1 of the hind legs was elevated from the treadmill, thus ensuring that the opposite foot/paw would be firmly planted against the treadmill belt allowing clear profiles of the planted foot. In total, 3 of the 6 frames were taken where the injured foot was planted, and 3 where the uninjured foot was planted. All the images were edited using Pixelmator Pro (version 1.6.1 Magenta, Vilnius, Lithuania). The exposure of the captured screenshot was increased to 80%, contrasted to 100% and black pointed to 100%. The image was then sharpened with the radius increased to 10.0 px and intensity to 100%, and then inverted to 100% intensity; this was done to make the margins of the paw in contact with the surface more prominent. The same setting was applied to all the images. Three separate measurements of experimental print length (i.e., the injured leg; EPL), normal print length (i.e., the uninjured leg; NPL), experimental toe spread (ETS) and normal toe spread (NTS) were obtained for each video, and then the mean of each measurement was recorded. SFI was then calculated from the mean of the measurements according to the formula [30]:

SFI = 118.9(ETS − NTS)/NTS − 51.2(EPL − NPL)/NPL − 7.5

### 2.4. Electrophysiological Assessment

Six weeks following surgery, the behavioral study was terminated. Prior to animal euthanasia, mice were anesthetized with 2–2.5% isoflurane. Nerve conduction velocity (NCV) was measured (Cadwell 6200A, Cadwell Laboratories, WA, USA) during nerve exposure of both the injured and uninjured legs based on previous reports [31,32]. Both intra-animal (comparing experimental limb to contralateral normal limb) and inter-animal studies were performed and analyzed. Motor latencies, conduction velocities and amplitudes were recorded. Customized bipolar hook electrode was used for stimulation of the proximal sciatic nerve just above the sciatic notch, and evoked compound muscle action potential (CMAP) activity was recorded from the lateral gastrocnemius muscle via 2 single needle electrodes (Ambu Neuroline Subdermal 12 × 0.40 mm, Ambu A/S Ballerup, Denmark) (Figure 1f). Suprathreshold stimulation with 1 mV consistently evoked stable CMAP responses. Distance from stimulation site to recording site was documented. The time of onset of the CMAP waveform divided by the distance between the stimulator and the recording electrode was used to generate NCV for each leg in each animal.

### 2.5. Histological Assessment

Following the collection of electrophysiological data, mice were euthanized by cervical dislocation. At this point, the sciatic nerves were isolated and stored in 4% paraformaldehyde overnight. The following morning, the nerves were washed in phosphate-buffered saline and placed in sucrose for 2 weeks at 4 °C. Nerves were then embedded in optimal cutting temperature compound and sectioned on a cryostat at a thickness of 10µm longitudinally. Sections were stained with neurofilament heavy chain (NF-H, 1:1000, Biolegend) and Glut1 (1:500, Sigma-Aldrich, St. Louis, MO, USA) to assess the axons and the perineurium [33,34], respectively. Finally, sections were counterstained with DAPI nuclear staining (1:2000) for 5 min and slides were covered with fluorescent mounting medium (FluorSave Reagent, Millipore) and coverslips followed by nail polish application at coverslip edges. Sections were examined in a blinded fashion, initially with a fluorescence microscope (Olympus BX51; Olympus, Japan) and imaged with a slide scanner (Olympus VS110-S5 Slidescanner, Japan). Nerves were assessed qualitatively for potential disruption of the perinurium and axons, as well as potential NIC formations. Identical immunohistochemical procedures were conducted in the exploratory and acute stages of injury model design.

### 2.6. Muscle Weight Assessment

Gastrocnemius and the soleus muscles of both the injured and uninjured legs were isolated, and their combined weight was recorded for each leg on a calibrated scale.

### 2.7. Statistics

GraphPad Prism version 9.3.1 for macOS (GraphPad Software, San Diego, CA, USA) was used for all statistical analyses. For comparisons between two groups (i.e., NCV, calf muscle weight), one-tailed unpaired *t*-tests were performed. For assessment of the data overtime (i.e., von Frey, SFI, gait analysis), two-way repeated measures ANOVAs were used with post-hoc analysis of differences between injury types assessed by Tukey’s HSD multiple comparisons test. Significance was determined as a *p*-value less than or equal to 0.05.

## 3. Results

### 3.1. Histology

**Figure 3:**
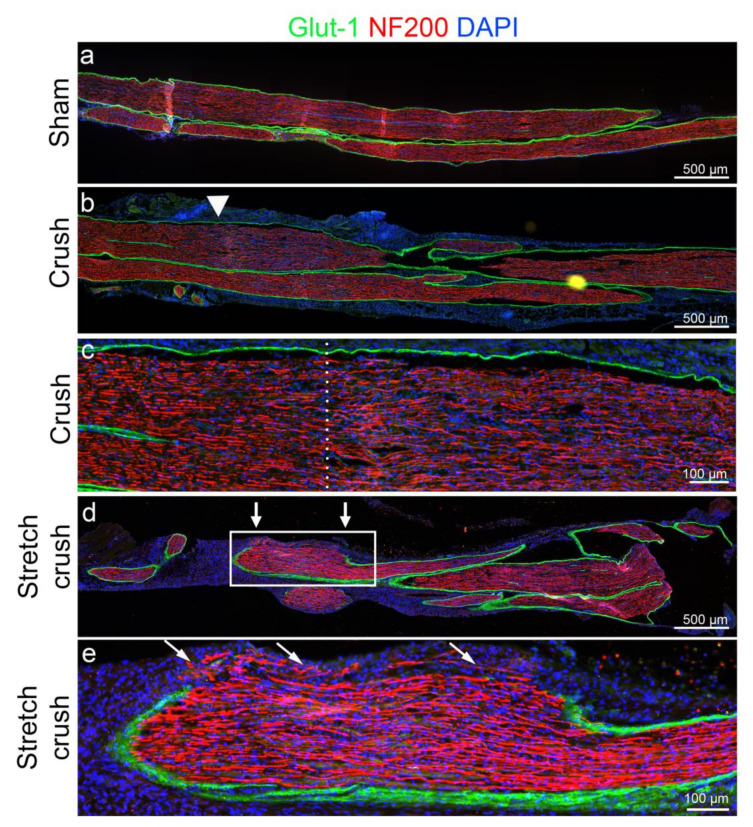
Histological comparison of acute six-day sciatic nerve injuries. (**a**) One-week uninjured sham nerve. (**b**) Crush injured nerve—note intact perineurium (Glut1, a marker of perineurium); yet, increased cellularity and potential inflammation related to Wallerian degeneration at and just beyond the crush site (white arrowhead). (**c**) Magnified crush-injury site of (**b**), dotted line marks the crush epicenter. Note again intact perineurium and the intense localization of nuclei (DAPI) distal (right of dotted line) to the crush site. In addition, thinned out disarrayed axons, compared to proximal to the crush zone, are evident. (**d**) Stretch–crush injured nerve. Note perineurium disruption at the injury site (between white arrows), significant nerve swelling and increased cellularity supporting an NIC development. (**e**) Magnified stretch–crush injury of (**d**), showing perineurial disruption as seen by Glut1 disruption, disarrayed axonal architecture with increased cellularity and several axons evident beyond the perineurial border—forming an NIC. NIC, neuroma in continuity.

**Figure 4:**
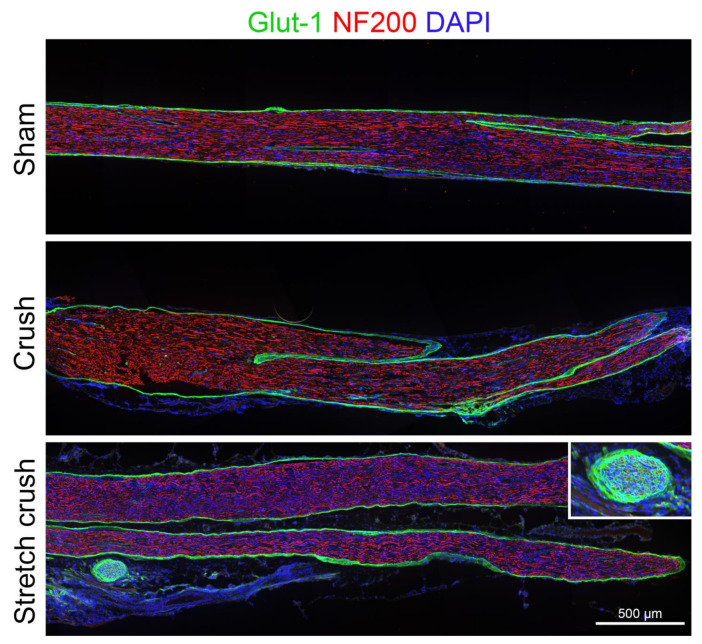
Six-week injury model comparison. Nerve microarchitecture is restored with intact perineurium in both injury types. Crush sites are not clearly identified in crush-only nerve, as markings were not clearly visible at time of sectioning. In the stretch–crush nerve, note the stained perineurial cells encompassing axons at injury site (insert), consistent with prior perineurium disruption (Figure 3e) and axonal regeneration intertwined with perineurial cell proliferation, findings consistent with an NIC injury. NIC, neuroma in continuity.

Qualitative analyses were performed for nerve histology. At week one post-injury, histology showed clear axonal architecture disarray, focal perineurial disruption with escape of axons beyond the perineurial boundary and swelling pathognomonic of a NIC [9] in the stretch–crush group. In contrast, in the crush-only group, the perineurium was intact with axonal disruption in the zone beyond the crush, along with increased cellularity, suggesting inflammatory infiltrate related to Wallerian degeneration [8] within fascicles (Figure 3). At the six-week endpoint, following the behavioral stage, histological analysis was carried out (Figure 4). The injury site was not readily identifiable in crush-only nerves. However, in stretch–crush nerves, axons were noted to be encompassed within perineurium and at times intertwined within (Figure 4 insert), a disrupted axonal growth pattern not evident in crush-only nerves [8].

### 3.2. Behavioral Assessments: Von Frey Monofilament Test, Gait Analysis and SFI

#### 3.2.1. Von Frey Monofilament Testing

**Figure 5:**
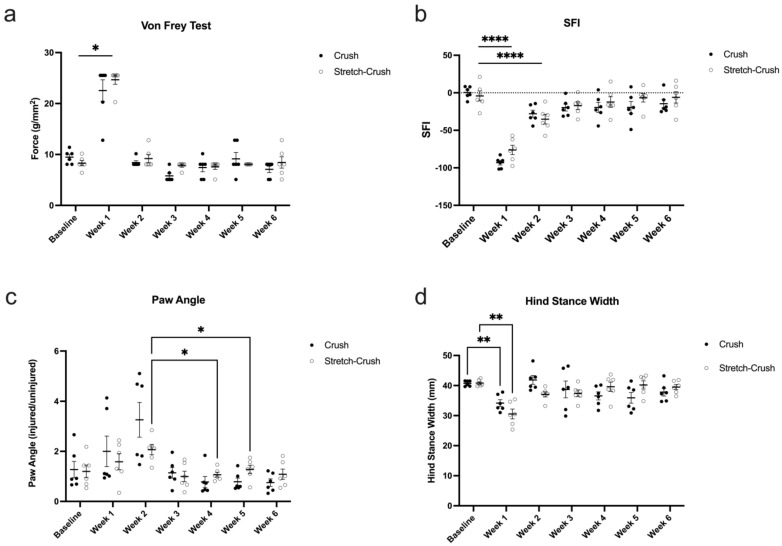
Behavioral and VGL metrics evaluated over six weeks. Results of crush-injured nerves compared to stretch–crush injured nerves and contralateral uninjured nerves (sham). (**a**) Von Frey test shows significant differences at week one in both groups compared to baseline, with a return towards baseline thereafter. (**b**) SFI follows a similar temporal profile of recovery as (**a**) with statistically significant measurements for both groups at week one and two compared to baseline. For HPA (**c**), measured as the ratio of the injured leg’s paw angle over the uninjured leg’s paw angle as assessed by VGL between injury models and across time, there was a surprising peak at week two. No significant differences between injury models were demonstrated; there was a trend, but nonsignificant interaction, between injury type and time (stretch–crush at week two compared to week four and five, *p* = 0.0672). (**d**) HSW analysis as assessed by VGL between injury models and across time showed significant differences for both groups at first week compared to baseline, with a similar kinetics of recovery over time, as the von Frey test (**a**) with return to baseline thereafter. Statistics were performed using a two-way repeated measures ANOVA. Error bars represent standard error of the mean (SEM). * = *p* < 0.05, ** = *p* < 0.01, **** = *p* < 0.0001. ANOVA, analysis of variance; HPA, hind paw angle; HSW, hind stance width; NCV, nerve conduction velocity; SFI, sciatic functional index; VGL, Visual Gait Lab.

Both injury types followed a similar post-injury timeline, with increased paw withdrawal thresholds corresponding to a decreased trend in sensation occurring in the first week after injury and returning to baseline levels by week two (Figure 5). A two-way repeated measures ANOVA revealed no significant differences between injury models (*F*(6, 60) = 1.131; *p* = 0.3558; *N* = 6 per group).

#### 3.2.2. SFI

Unlike von Frey monofilament testing, two-way repeated measures ANOVA revealed a significant interaction between injury type and time (*F*(6, 60) = 2.426; *p* < 0.05; *N* = 6 per group). Post hoc analysis revealed that in the crush group, SFI differed significantly from baseline for the first five weeks post-injury, whereas in the stretch–crush condition, SFI only differed significantly from the baseline at week one and two, post-injury (Figure 5).

#### 3.2.3. Visual Gait Lab Analysis

Using VGL, videos were analyzed for hind paw angle (HPA)—measured as the angle, in degrees, between the two markers of each paw and the central body line (Figure 5c). Similarly, hind stance width (HSW) was calculated based on incorporation of the number of frames collected between full stance and the end of a full stride (Figure 5d). For HPA, a two-way repeated measures ANOVA revealed no significant differences between injury models; however, surprisingly, HPA peaked unexpectedly at week two, and showed a trend, but nonsignificant interaction, between injury type and time, *F*(6, 60) = 2.093; *p* = 0.067; *N* = 6 per group (stretch-crush at week two compared to week four and five). These findings were most likely attributed to the reduced variability (see SEM bars in week two) in the stretch–crush injury at two weeks post-injury compared to the crush group. In general, it seems that the animals had an elevated paw angle for the first two weeks post-injury, regardless of injury type, eventually retuning to baseline levels by week three. HSW was also assessed by VGL, and a two-way repeated measures ANOVA showed a significant interaction between injury and time (*F*(6, 60) = 3.961; *p* < 0.01; *N* = 6 per group; Figure 5c). Post hoc analysis showed that in both injury groups, there was a significant decrease in HSW at week one compared to baseline. The interaction between injury and time is most likely attributed to the recovery of the week one decrease in HSW, whereas in the crush group, the HSW rebounds to baseline levels at week two, before gradually going back down again, subtly; however, in the stretch–crush group, following week one there is a gradual return of HSW to baseline levels over the remaining five-week recovery period. For complete mathematical formulation and calculation, including detailed frame analysis, please refer to Fiker et al., 2020 [21].

### 3.3. Electrophysiological and Muscle Weight Assessment

**Figure 6:**
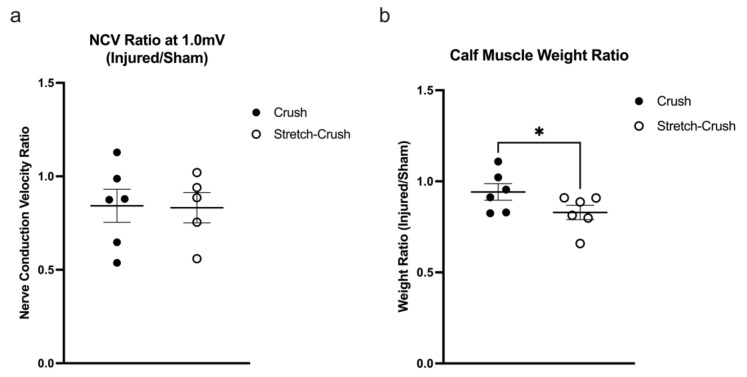
Electrophysiological and muscle weight assessment. (**a**) Comparison of NCV of crush and stretch–crush injured legs relative to contralateral uninjured legs (sham) at the six-week study termination point. Note that the average NCV ratios remain <1, but do not differ significantly between injury conditions. (**b**) Calf muscle weight comprised of the combined weight of the gastrocnemius and soleus muscles measured at the study termination point at six weeks following injury and converted into a ratio of the injured leg over the uninjured leg. Reduced calf muscle weight in the stretch–crush condition compared to the crush alone shows significant difference (*p* < 0.05). Statistics were performed using a one-tailed unpaired *t*-test. Error bars represent standard error of the mean (SEM). * = *p* < 0.05. NCV, nerve conduction velocity.

Interestingly, when NCV were assessed following a 1.0 mV (suprathreshold) stimulation at six weeks post-injury from injured and uninjured legs from both injury models, there was no significant difference between models, as assessed by a one-tailed unpaired *t*-test, *t*(9) = 0.08548; *p* = 0.4669 (Figure 6a). One animal had to be excluded from the stretch–crush group due to an inability to obtain a consistent recording (crush group: *M* = 0.8425, *SD* = 0.2175, *N* = 6; stretch–crush: *M* = 0.8320, *SD* = 0.1805, *N* = 5). NCVs did not return quite to baseline at week six, as the conduction in the contralateral normal leg remained slightly greater than the experimental one (ratio < 1). Following the electrophysiological measure, the calf muscle (i.e., combined weight of the gastrocnemius and the soleus muscles) of both the injured and uninjured legs was isolated and weighed (Figure 6b). The weights were then converted into a ratio of the injured leg over the uninjured leg. A one-tailed unpaired *t*-test was performed, and revealed a significant difference between the injury models (*t*(10) = 1.879; *p* < 0.05) with a reduced calf muscle weight in the stretch–crush condition (*M* = 0.8291, *SD* = 0.0963, *N* = 6) compared to crush alone (*M* = 0.9418, *SD* = 0.1110, *N* = 6).

## 4. Discussion

While a significant understanding of both the molecular processes of peripheral nerve regeneration as well as injury mechanism and histopathological processes has occurred in recent years [6,7,35,36,37,38,39]; a single ‘gold-standard’ verified and repeatable injury model is still lacking. Furthermore, models have repeatedly used rodents as their primary investigative purposes for multiple reasons, including relatively low costs, even though they have several limitations, such as small size, relatively short regeneration distance, specific neurobiological regenerative profile, faster regenerating capacity and bifascicular nerves in contrast to polyfascicular human nerves [10,40]. Even so, attempting to prove translation into larger non-human primates [41] as well as clinical treatment heavily relies on these smaller animal models. Moreover, a well-developed and accepted rodent nerve injury model would harness the powerful tool of genetic engineering to decipher molecular and mechanistic pathways which may underlie the development of new therapies. Rodents models have been the mainstay for investigation, owing to their resilience and effective peripheral nerve regeneration [9]. Both in vitro and in vivo [42,43] nerve investigations along with functional and behavioral studies have employed this approach; yet, a large deficit in our knowledge still surrounds the understanding of the injury mechanism itself.

In a recent set of publications, Mahan et al. detailed specific injury conditions in rodents [8,11,15,44] presenting a rapid stretch injury rodent nerve trauma model to better understand tissue biomechanical responses involved in injuries concentrating on biomechanical and histological analysis of rapid-stretch injuries. Yet, a significant account of their work centered around rat models, while reproducibility and evaluation of similar injury models in mice is still, currently, limited.

Herein, we have established a standardized stretch–crush injury model in mice that follows our groups prior validated crush-injury model in rats [9,45]. The current model produces a somewhat more severe injury than an axonotmetic Sunderland grade 2 injury in the crush-only group, at least initially after injury, which is evident in histological examinations at one week post-injury and supported by behavioral studies. While subtle and nonsignificant behavioral differences were observed between the two injury groups over the remainder of the follow-up period, similar recovery of behavioral parameters in both injury groups to baseline were evident by week six. Only the final calf muscle weight measurements showed apparent significant differences.

Our attempt to create a novel NIC model in mice using a reproducible injury has raised certain questions. Our observation of initial neurological deficit as maximal over a one- to two-week frame, but under three weeks, as observed in a large longitudinal study [46], is consistent with prior reports [8,47,48,49]. Yet, the overall similarity of behavioral recovery kinetics in the two injury groups was unexpected, given that we evidently produced a more severe injury using the stretch–crush method. We are left, however, with several reassuring observations. Wet muscle weight, expected to return to baseline following a crush injury, supports endoneurial architecture preservation, which will allow the successful guidance of axons to their previous target [8,50] with reinnervation. Our apparent significant difference between the two groups is in keeping with a more severe injury occurring in our stretch–crush group compared to the crush alone. This latter finding might also be supported by the notable perineurial entwined axons seen at the six-week histology, suggesting an NIC with complete functional recovery, albeit not histologically visible [8,44]. Furthermore, it is consistent with dynamic fast regeneration and plasticity in certain injury mechanisms [8] in young adult mice physiologically overcoming NIC formation. Finally, the stretch–crush injury in mice seems to be less severe than that created in rats, as the latter has more durable behavioral deficits and evidence of axonal attrition and misdirection [9,45]. There are indeed plasticity and peripheral regeneration differences between rats and mice [51,52,53,54], species differences may account for the findings of some differences in the recovery from stretch–crush in rats versus mice.

Spatiotemporal gait analysis provides experimental animal paw movement in both space and time, including several parameters such as stride length, step width, limb phase and footprint characteristics [55]. Initial gait investigations using ink prints had inherently suffered from cumbersome technical analysis and shortcoming lacking velocity calculations [56]. Later semi-automated treadmill devices including CatWalk (Noldus, Wageningen, The Netherlands), GaitScan (CleverSys Reston, VA), TreadScan (CleverSys Reston, VA) and our currently used DigiGait system use image pixel intensity contrast to distinguish paws from background, and are limited by inconsistent stride measurements, reproducibility, operator experience and operator bias [28,57,58]. The use of a deep-learning approach for PNI gait analysis, to our knowledge, has not been attempted before. We have chosen to concentrate on two main parameters of VGL in our current mouse PNI model, namely, HPA and HSW. Our results demonstrated that the degree of HPA trends at the first two weeks to favor the animals’ injured leg, compared to the last two weeks, when recovery is expected to occur (Figure 3). HPA supports a limping gait [59]—herein, mostly apparent through the first two weeks following injury—and, interestingly, did not follow the exact trend as HSW. HSW followed a similar post-injury trajectory as the standard manual assessment and provided, for the first time, delineation of VGL parameters that mirror recovery kinetics compared to standard SFI assessment. HSW in millimeters was demonstrated to be significantly smaller in both groups at the first week following injury, compared to baseline, and between the first and second weeks.

It is important to note that VGL can calculate HPA and HSW internally by comparing assigned marker positioning to multiple pre-defined anatomical landmarks (i.e., nose, tail base and abdominal-pelvic boundary) of the investigated animal, in an animal-by-animal fashion and in comparison, to ipsilateral and contralateral front paws and animal body tracking. This latter point allows us to demonstrate previously uninvestigated observation metrics and, furthermore, allows us to incorporate average stance, swing and stride over time in milliseconds. These parameters were chosen for being representative in both static and dynamic output measures, as they have been reported in both Parkinsonian and amyotrophic lateral sclerosis gait-to-gait analysis reports [59,60,61], and have provided us with quantifiable results in comparison with averaged baseline mouse gait. Furthermore, we believe they can harbor insight into the above previously overlooked observations and can both augment and possibly replace SFI as the sole investigation parameter of injury in future works.

Deep neural networks (DNNs) are computational algorithms consisting of simple units, organized in layers, and then serially stacked to form ‘deep networks’ using a Python programming package. The connections between the units are trained on datasets and, therefore, learn to extract information from raw data in order to solve tasks [62]. For these networks to compute smaller datasets, as in this work, transfer learning (i.e., using a network trained on one task to perform another) was performed and established in DLC [20], on which VGL was developed [21], allowing the user to use only a few hundred annotated images to achieve gait tracking. Furthermore, DLC has been shown to reach human labeling accuracy [22], yet concerns regarding adaptation of these CNN to model human final judgement object recognition are still debatable [63].

Using VGL attempts to help us answer previous limitations of classic gait studies. SFI has notoriously been known to require several trials to obtain representative recordings for analysis and a cumbersome technique of training [12,17,64], especially when significant hesitancy is encountered by the animal investigated [65,66,67]. These limitations can hopefully be tailored and adjusted through a trained deep-learning interface to assess PNI more specifically in the future. Gait analysis carried out through VGL was herein verified independently in a blinded nonbiased fashion through analysis and comparison to SFI and print length. In a comparative analysis, however, we could not find similar trends in paw length compared through VGL to human observers [20,22] and we have not presented this herein. While considered a determinantal reason for utilization of VGL and DNNs, we still believe tailoring proper training data necessary for DNN to function optimally [62] may provide superior, closer to ‘gold standard’ results, in PNI models’ gait tracking.

Impairment of NCV is common in neurodegenerative and neuroinflammatory conditions, while reduced nerve conduction velocity resulting from axonopathy or demyelination strongly affects functional recovery [18,68]. Furthermore, NCV is closely related to fiber diameter and myelin thickness [69]. Interestingly, there were no significant differences in NCV at the termination of the experiment, and while we were expecting certain differences to be apparent at that timepoint or earlier, the latter were not performed and should be considered in future studies. Finally, although NCV ratio averages recorded at the gastrocnemius muscle at six weeks’ time did not return completely to baseline and remained <1, they did not differ significantly between injury conditions. Thus, we cannot exclude that different innervation pathways and/or formation of the neuromuscular junction have indeed occurred during recovery—implying different muscle innervation and formation might play a key role [70]. Indeed, we postulate that ongoing peripheral plasticity in the more severe NIC may be contributing to the findings. Specifically, the lack of full maturation of neuromuscular junctions and robust muscle reinnervation at six weeks in the stretch–crush versus the simple stretch–crush may have contributed to the divergence in the NCV and muscle weight data at study termination in the former. It is likely that if later timepoints (8–12 weeks) had been evaluated, the two injury groups would have been indistinguishable.

Our study has several limitations. First, the behavioral study was performed in a group of young animals and might have shown more significant differences between the two on a larger sample set of older animals. Whether the results would be impacted by the sex of the animal is also unknown. Secondly, NCV which were performed at the study termination point have not shown any significant differences between the two investigated groups; yet, like early time-point histological results and behavioral studies, NCV might have been more remarkable and informative if performed in an ongoing serial fashion. Finally, the nature of utilizing VGL and deep-learning platform parameters (i.e., HPA and HSW) through a pilot study requires us, as previously mentioned, to properly tailor this tool for hindlimb PNI gait analysis in a larger dataset.

## 5. Conclusions

Stretch–crush injury achieved a more reproducible and constant injury compared to crush-only injuries, with at least a Sunderland grade 3 injury (perineurial interruption) in histological samples one week post-injury in the former. However, serial behavioral outcomes were comparable in the two injury groups, with similar kinetics of recovery by von Frey testing, SFI and VGL parameters. The latter were reported for the first time in rodent PNI and their use in the future appears promising. Overall, NCV findings further support regeneration and return to baseline in both injuries, while outstanding final muscle weight comparisons did provide significant differences between injury models, suggesting certain physiological differences might have been overlooked. Regardless, our data suggests that the stretch–crush injury, that is more akin to PNS injuries in humans, is a robust model in mice and, thus, may be a better model to pursue in future PNS regeneration studies.

## Figures and Tables

**Table 1:** Description of mice used for various stages of the study.

Experimental Stage	Number	Strain
Exploratory	19	Varying
Acute	4	C57/Bl6
Behavioral	12	129S6/SvEvTac

## Data Availability

Not applicable.
